# Supplementary material for: Identification of 2,4-Diaminoquinazoline Derivative as a Potential Small-Molecule Inhibitor against Chikungunya and Ross River Viruses
Source: Viruses. 2023 Oct 31;15(11):2194. doi: 10.3390/v15112194 (PMC10674894; doi:10.3390/v15112194)
Supplement: Supplementary file 1 [file viruses-15-02194-s001.zip › viruses-2629895-supplementary.pdf]

Supplementary Materials for

Identification of 2, 4 -Diaminoquinazoline Derivative as a Potential Small-Molecule Inhibitor against Chikungunya and Ross River Viruses

This file includes:

Table S1

Table S1: List of in-house small molecules screened for CHIKV

DCR code	Structure	IUPAC name	Mol Formula	Mol Wt (Da)	MNTD* (μM)	CPE**	% Cell viability***	Reference
**DCR1**	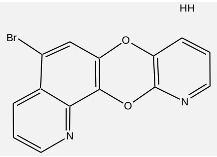	5-Bromo-7,12-dioxa-1,11-diaza-benzo[a]anthracene	$C_{14}H_7BrN_2O_2$	315.12	50	++	72	BN Acharya et al, Med Chem Res, 2008, 17, 487-494
**DCR2**	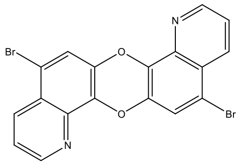	10,21-dibromo-2,13-dioxa-5,16-diazapentacyclo[12.8.0.$0^{3,12}$.$0^{4,9}$.$0^{15,20}$]docosa-1(14),3(12),4,6,8,10,15(20),16,18,21-decaene	$C_{18}H_8Br_2N_2O_2$	444.07	50	++	63	BN Acharya et al, Med Chem Res, 2008, 17, 487-494
**DCR3**	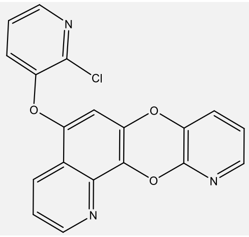	7-[(2-chloropyridin-3-yl)oxy]-5,12-dioxa-1,11-diazatetraphene	$C_{19}H_{10}ClN_3O_3$	363.754	50	++	69.9	BN Acharya et al, Med Chem Res, 2008, 17, 487-494
**DCR4**	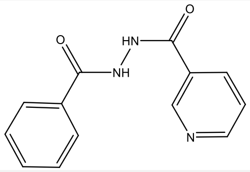	N'-benzoylnicotinohydrazide	$C_{13}H_{11}N_3O_2$	241.24	31.25	++++	15	
**DCR5**	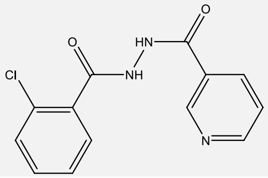	N'-(2-chlorobenzoyl)nicotinohydrazide	$C_{13}H_{10}ClN_3O_2$	275.69	31.25	++++	13.49	
**DCR6**	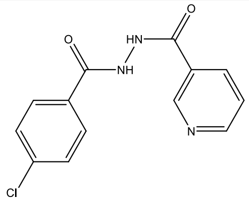	N'-(4-chlorobenzoyl)nicotinohydrazide	$C_{13}H_{10}ClN_3O_2$	275.69	31.25	++++	16.98	
**DCR7**	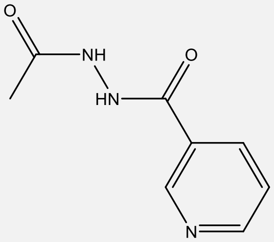	N'-acetylnicotinohydrazide	$C_8H_9N_3O_2$	179.17	62.5	++++	20.31	

**DCR8**	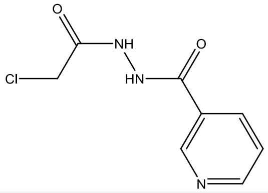	N'-(2-chloroacetyl)nicotinohydrazide	$C_8H_8ClN_3O_2$	213.62	31.25	++++	13.8
**DCR9**	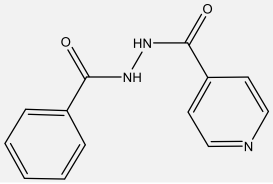	N'-benzoylisonicotinohydrazide	$C_{13}H_{11}N_3O_2$	241.24	31.25	++++	9.92
**DCR10**	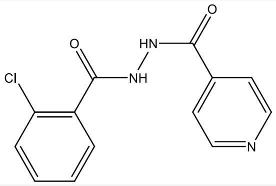	N'-(2-chlorobenzoyl)isonicotinohydrazide	$C_{13}H_{10}ClN_3O_2$	275.69	31.25	++++	21.19
**DCR11**	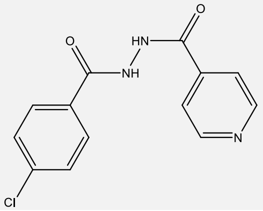	N'-(4-chlorobenzoyl)isonicotinohydrazide	$C_{13}H_{10}ClN_3O_2$	275.69	31.25	++++	22.85
**DCR12**	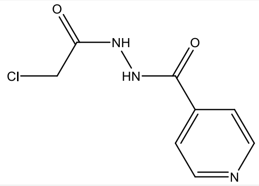	N'-(2-chloroacetyl)isonicotinohydrazide	$C_8H_8ClN_3O_2$	213.62	31.25	++++	16.34
**DCR13**	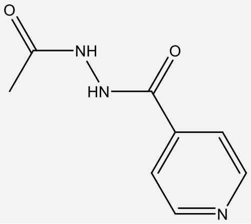	N'-acetylisonicotinohydrazide	$C_8H_9N_3O_2$	179.17	62.5	++++	15.07
**DCR14**	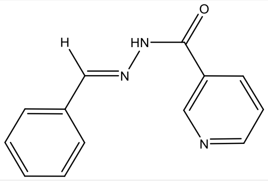	(E)-N'-benzylidenenicotinohydrazide	$C_{13}H_{11}N_3O$	225.24	31.25	++++	22.93
**DCR15**	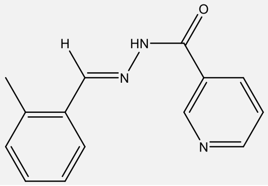	(E)-N'-(2-methylbenzylidene)nicotinohydrazide	$C_{14}H_{13}N_3O$	239.27	31.25	++++	14.76

**DCR16**	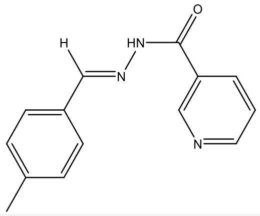	(E)-N'-(4-methylbenzylidene)nicotinohydrazide	$C_{14}H_{13}N_3O$	239.27	31.25	++++	18.49
**DCR17**	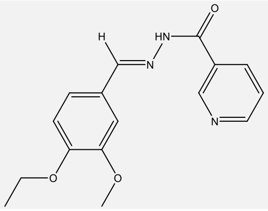	(E)-N'-(4-ethoxy-3-methoxybenzylidene) nicotinohydrazide	$C_{16}H_{17}N_3O_3$	299.32	31.25	++++	0.1
**DCR18**	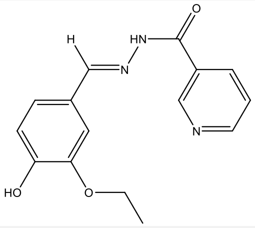	(E)-N'-(3-ethoxy-4-hydroxybenzylidene)nicotinohydrazide	$C_{15}H_{15}N_3O_3$	285.29	31.25	++++	15.39
**DCR19**	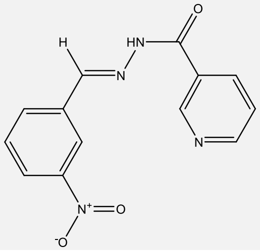	(E)-N'-(3-nitrobenzylidene)nicotinohydrazide	$C_{13}H_{10}N_4O_3$	270.24	62.5	++++	20.39
**DCR20**	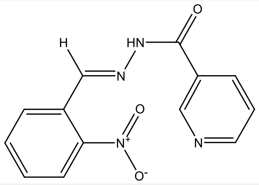	(E)-N'-(2-nitrobenzylidene)nicotinohydrazide	$C_{13}H_{10}N_4O_3$	270.24	31.25	++++	22.69
**DCR21**	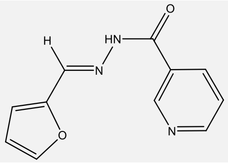	(E)-N'-(furan-2-ylmethylene)nicotinohydrazide	$C_{11}H_9N_3O_2$	215.20	62.5	++++	0
**DCR22**	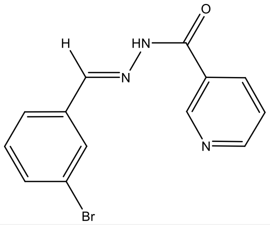	(E)-N'-(3-bromobenzylidene)nicotinohydrazide	$C_{13}H_{10}BrN_3O$	304.14	15.6	++++	19.28
**DCR23**	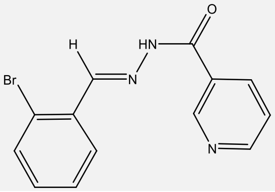	(E)-N'-(2-bromobenzylidene)nicotinohydrazide	$C_{13}H_{10}BrN_3O$	304.14	15.6	++++	28.01

**DCR24**	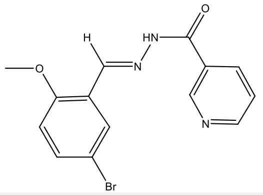	(E)-N'-(5-bromo-2-methoxybenzylidene) nicotinohydrazide	$C_{14}H_{12}BrN_3O_2$	334.16	15.6	++++	25.23
**DCR25**	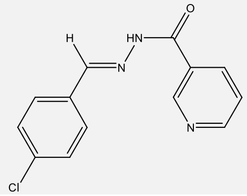	(E)-N'-(4-chlorobenzylidene)nicotinohydrazide	$C_{13}H_{10}ClN_3O$	259.69	62.5	++++	30.15
**DCR26**	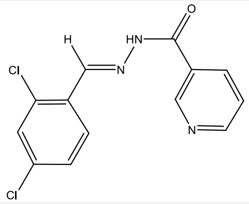	(E)-N'-(2,4-dichlorobenzylidene)nicotinohydrazide	$C_{13}H_9Cl_2N_3O$	294.13	31.25	++++	26.11
**DCR27**	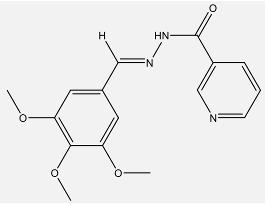	(E)-N'-(3,4,5-trimethoxybenzylidene)nicotinohydrazide	$C_{16}H_{17}N_3O_4$	315.32	31.25	++++	21.26
**DCR28**	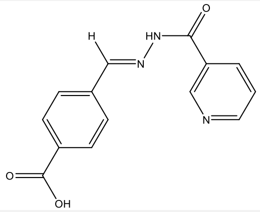	(E)-4-((2-nicotinoylhydrazono) methyl)benzoic acid	$C_{14}H_{11}N_3O_3$	269.25	62.5	++++	15.63
**DCR29**	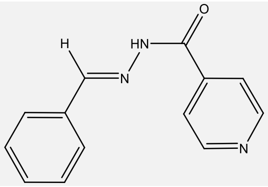		$C_{13}H_{11}N_3O$	225.24	62.5	++++	20.79
**DCR30**	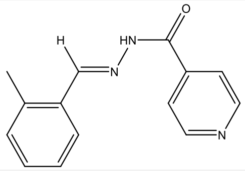	(E)-N'-(2-methylbenzylidene)isonicotinohydrazide	$C_{14}H_{13}N_3O$	239.27	62.5	++++	23.8
**DCR31**	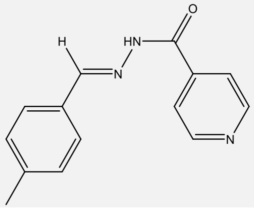	(E)-N'-(4-methylbenzylidene)isonicotinohydrazide	$C_{14}H_{13}N_3O$	239.27	62.5	++++	1.03

**DCR32**	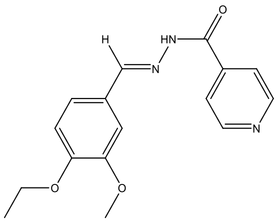	(E)-N'-(4-ethoxy-3-methoxybenzylidene)isonicotinohydrazide	$C_{16}H_{17}N_3O_3$	299.32	31.25	++++	6.03
**DCR33**	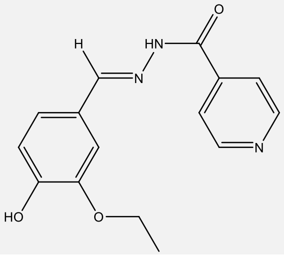	(E)-N'-(3-ethoxy-4-hydroxybenzylidene)isonicotinohydrazide	$C_{15}H_{15}N_3O_3$	285.29	62.5	++++	0
**DCR34**	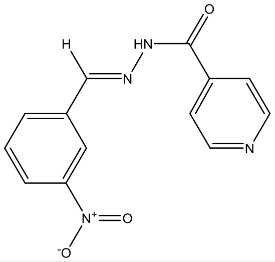	(E)-N'-(3-nitrobenzylidene)isonicotinohydrazide	$C_{13}H_{10}N_4O_3$	270.24	62.5	++++	6.42
**DCR35**	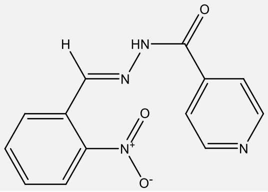	(E)-N'-(2-nitrobenzylidene)isonicotinohydrazide	$C_{13}H_{10}N_4O_3$	270.24	62.5	++++	9.84
**DCR36**	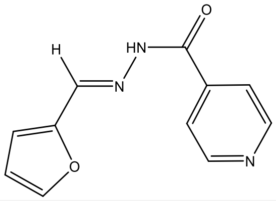	(E)-N'-(furan-2-ylmethylene)isonicotinohydrazide	$C_{11}H_9N_3O_2$	215.20	62.5	++++	5
**DCR37**	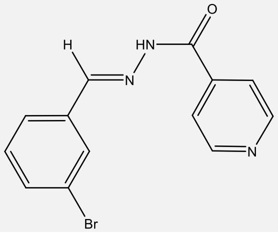	(E)-N'-(3-bromobenzylidene)isonicotinohydrazide	$C_{13}H_{10}BrN_3O$	304.14	31.25	++++	14.36
**DCR38**	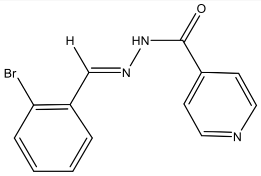	(E)-N'-(2-bromobenzylidene)isonicotinohydrazide	$C_{13}H_{10}BrN_3O$	304.14	31.25	++++	25.1

**DCR39**	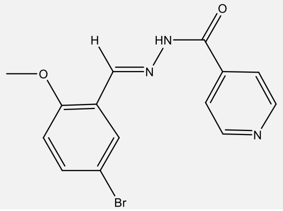	(E)-N'-(5-bromo-2-methoxybenzylidene)isonicotinohydrazide	$C_{14}H_{12}BrN_3O_2$	334.16	31.25	++++	3.81
**DCR40**	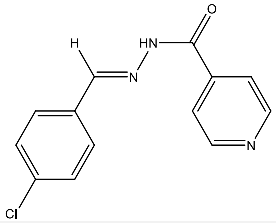	(E)-N'-(4-chlorobenzylidene)isonicotinohydrazide	$C_{13}H_{10}ClN_3O$	259.69	31.25	++++	5.23
**DCR41**	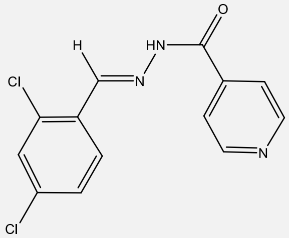	(E)-N'-(2,4-dichlorobenzylidene)isonicotinohydrazide	$C_{13}H_9Cl_2N_3O$	294.13	31.25	++++	35.79
**DCR42**	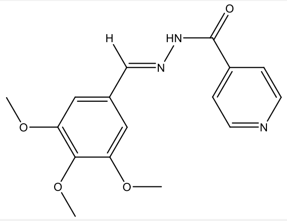	(E)-N'-(3,4,5-trimethoxybenzylidene)isonicotinohydrazide	$C_{16}H_{17}N_3O_4$	315.32	31.25	++++	12.22
**DCR43**	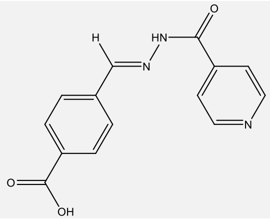	(E)-4-((2-isonicotinoylhydrazono)methyl)benzoic acid	$C_{14}H_{11}N_3O_3$	269.25	31.25	++++	16.82
**DCR44**	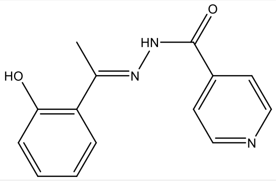	(E)-N'-(1-(2-hydroxyphenyl)ethylidene)isonicotinohydrazide	$C_{14}H_{13}N_3O_2$	255.27	31.25	++++	18.88
**DCR45**	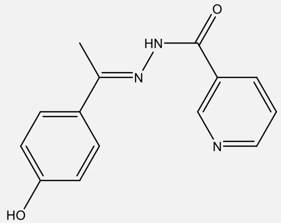	(E)-N'-(1-(4-hydroxyphenyl)ethylidene)nicotinohydrazide	$C_{14}H_{13}N_3O_2$	255.27	31.25	++++	15.55
**DCR46**	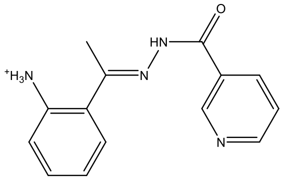	(E)-2-(1-(2-nicotinoylhydrazono)ethyl)benzenaminium	$C_{14}H_{15}N_4O$	255.29	31.25	++++	27.31

**DCR47**	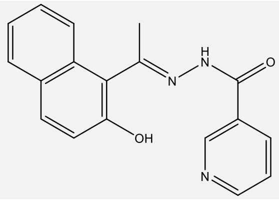	(E)-N'-(1-(2-hydroxynaphthalen-1-yl)ethylidene)nicotinohydrazide	$C_{18}H_{15}N_3O_2$	305.33	31.25	++++	6.42
**DCR48**	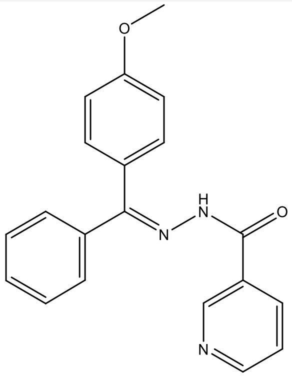	(Z)-N'-((4-methoxyphenyl)(phenyl)methylene)nicotinohydrazide	$C_{20}H_{17}N_3O_2$	331.36	31.25	++++	20.87
**DCR49**	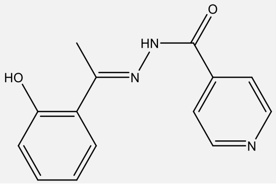	(E)-N'-(1-(2-hydroxyphenyl)ethylidene)isonicotinohydrazide	$C_{14}H_{13}N_3O_2$	255.27	62.5	+++	48.9
**DCR50**	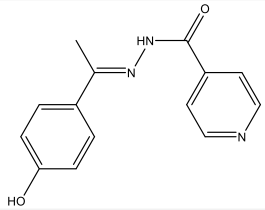	(E)-N'-(1-(4-hydroxyphenyl)ethylidene)isonicotinohydrazide	$C_{14}H_{13}N_3O_2$	255.27	62.5	++++	18.01
**DCR51**	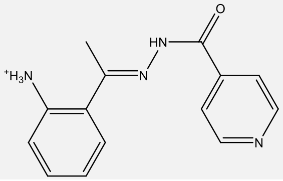	(E)-2-(1-(2-isonicotinoylhydrazono)ethyl)benzenaminium	$C_{14}H_{15}N_4O$	255.29	62.5	++++	-2.93
**DCR52**	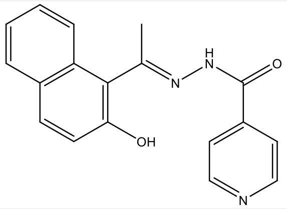	N'-[(1E)-1-(2-hydroxynaphthalen-1-yl)ethylidene]pyridine-4-carbohydrazide	$C_{18}H_{15}N_3O_2$	305.33	31.25	++++	30.87
**DCR53**	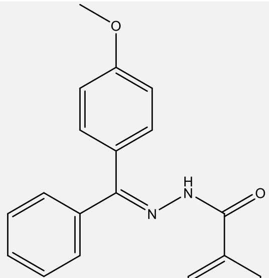	N'-[(1Z)-(4-methoxyphenyl)(phenyl)methylidene]pyridine-4-carbohydrazide	$C_{20}H_{17}N_3O_2$	331.36	31.25	++++	11.42

**DCR54**	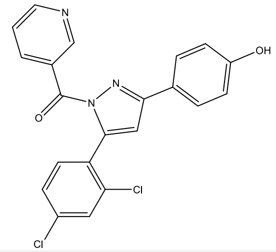	4-[5-(2,4-dichlorophenyl)-1-(pyridine-3-carbonyl)-1H-pyrazol-3-yl]phenol	$C_{21}H_{13}Cl_2N_3O_2$	410.25	15.62	++++	8.73	BN Acharya et al, Eu. J. Med.Chem, 2010, 45, 430-438
**DCR55**	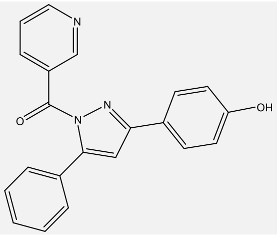	4-[5-phenyl-1-(pyridine-3-carbonyl)-1H-pyrazol-3-yl]phenol	$C_{21}H_{15}N_3O_2$	341.36	31.25	++++	17.69	BN Acharya et al, Eu. J. Med.Chem, 2010, 45, 430-438
**DCR56**	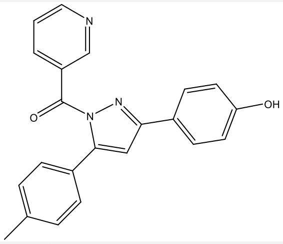	4-[5-(4-methylphenyl)-1-(pyridine-3-carbonyl)-1H-pyrazol-3-yl]phenol	$C_{22}H_{17}N_3O_2$	355.38	31.25	++++	24.2	BN Acharya et al, Eu. J. Med.Chem, 2010, 45, 430-438
**DCR57**	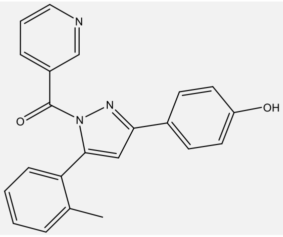	4-[5-(4-methylphenyl)-1-(pyridine-3-carbonyl)-1H-pyrazol-3-yl]phenol	$C_{22}H_{17}N_3O_2$	355.38	31.25	++++	24.84	BN Acharya et al, Eu. J. Med.Chem, 2010, 45, 430-438
**DCR58**	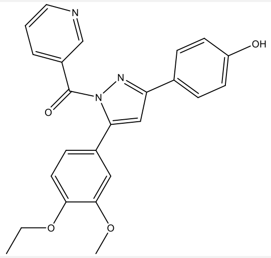	4-[5-(4-ethoxy-3-methoxyphenyl)-1-(pyridine-3-carbonyl)-1H-pyrazol-3-yl]phenol	$C_{24}H_{21}N_3O_4$	415.44	15.62	++++	8.17	BN Acharya et al, Eu. J. Med.Chem, 2010, 45, 430-438
**DCR59**	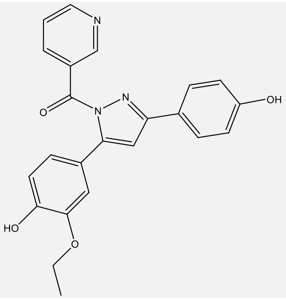	2-ethoxy-4-[3-(4-hydroxyphenyl)-1-(pyridine-3-carbonyl)-1H-pyrazol-5-yl]phenol	$C_{23}H_{19}N_3O_4$	401.41	15.62	+++	49.1	BN Acharya et al, Eu. J. Med.Chem, 2010, 45, 430-438

**DCR60**	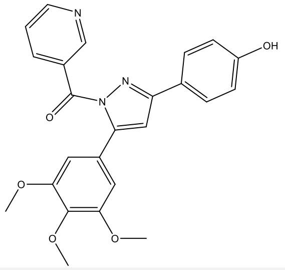	4-[1-(pyridine-3-carbonyl)-5-(3,4,5-trimethoxyphenyl)-1H-pyrazol-3-yl]phenol	$C_{24}H_{21}N_3O_5$	431.44	31.25	++++	-3.41	BN Acharya et al, Eu. J. Med.Chem, 2010, 45, 430-438
**DCR61**	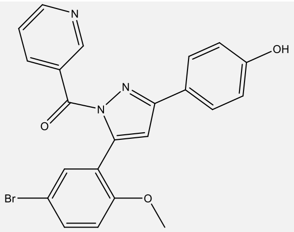	4-[5-(5-bromo-2-methoxyphenyl)-1-(pyridine-3-carbonyl)-1H-pyrazol-3-yl]phenol	$C_{22}H_{16}BrN_3O_3$	450.28	31.25	++++	12.22	BN Acharya et al, Eu. J. Med.Chem, 2010, 45, 430-438
**DCR62**	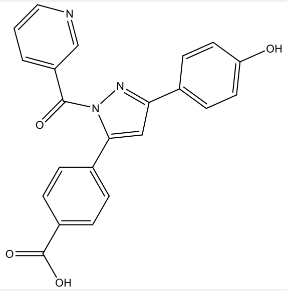	4-[3-(4-hydroxyphenyl)-1-(pyridine-3-carbonyl)-1H-pyrazol-5-yl]benzoic acid	$C_{22}H_{15}N_3O_4$	385.37	31.25	++++	-27.93	BN Acharya et al, Eu. J. Med.Chem, 2010, 45, 430-438
**DCR63**	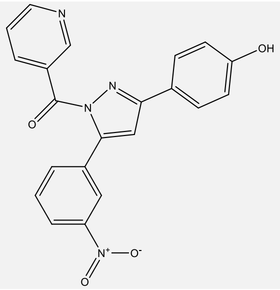	4-[5-(3-nitrophenyl)-1-(pyridine-3-carbonyl)-1H-pyrazol-3-yl]phenol	$C_{21}H_{14}N_4O_4$	386.36	31.25	++++	-4.2	BN Acharya et al, Eu. J. Med.Chem, 2010, 45, 430-438
**DCR64**	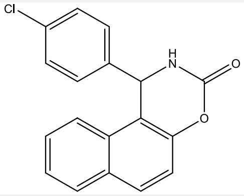	1-(4-chlorophenyl)-1H,2H,3H-naphtho[1,2-e][1,3]oxazin-3-one	$C_{18}H_{12}ClNO_2$	309.74	31.25	++++	-3.8	GBD Rao et al, Tetrahedron Lett. 2012, 53, 2741-2744
**DCR65**	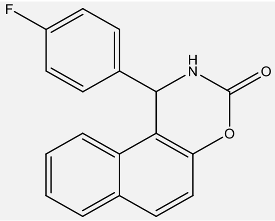	1-(4-fluorophenyl)-1H,2H,3H-naphtho[1,2-e][1,3]oxazin-3-one	$C_{18}H_{12}FNO_2$	293.29	53.27	++++	12.63	GBD Rao et al, Tetrahedron Lett. 2012, 53, 2741-2744

**DCR66**	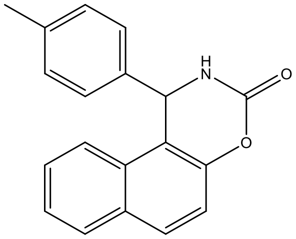	1-(4-methylphenyl)-1H,2H,3H-naphtho[1,2-e][1,3]oxazin-3-one	$C_{19}H_{15}NO_2$	289.32	54	++++	12.24	GBD Rao et al, Tetrahedron Lett. 2012, 53, 2741-2744
**DCR67**	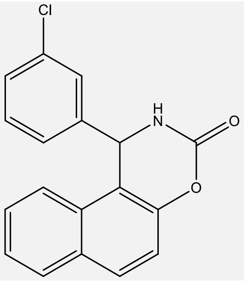	1-(3-chlorophenyl)-1H,2H,3H-naphtho[1,2-e][1,3]oxazin-3-one	$C_{18}H_{12}ClNO_2$	309.74	50.44	++++	10.94	GBD Rao et al, Tetrahedron Lett. 2012, 53, 2741-2744
**DCR68**	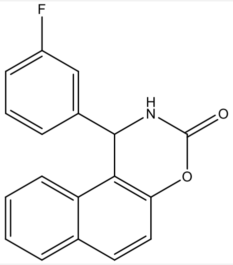	1-(3-chlorophenyl)-1H,2H,3H-naphtho[1,2-e][1,3]oxazin-3-one	$C_{18}H_{12}FNO_2$	293.29	53.27	++++	10.84	GBD Rao et al, Tetrahedron Lett. 2012, 53, 2741-2744
**DCR69**	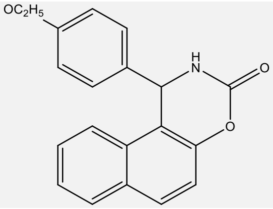	1-(4-ethoxyphenyl)-1H,2H,3H-naphtho[1,2-e][1,3]oxazin-3-one	$C_{20}H_{17}NO_3$	319.35	195.71	++++	7.61	GBD Rao et al, Tetrahedron Lett. 2012, 53, 2741-2744
**DCR70**	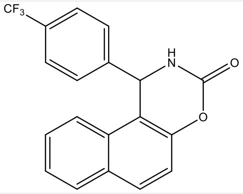	1-[4-(trifluoromethyl)phenyl]-1H,2H,3H-naphtho[1,2-e][1,3]oxazin-3-one	$C_{19}H_{12}F_3NO_2$	343.29	45.44	++++	10.59	GBD Rao et al, Tetrahedron Lett. 2012, 53, 2741-2744
**DCR71**	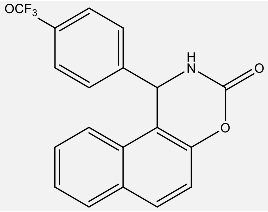	1-[4-(trifluoromethoxy)phenyl]-1H,2H,3H-naphtho[1,2-e][1,3]oxazin-3-one	$C_{19}H_{12}F_3NO_3$	359.29	43.42	++++	7.44	GBD Rao et al, Tetrahedron Lett. 2012, 53, 2741-2744
**DCR72**	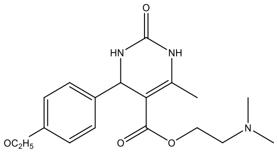	2-(dimethylamino)ethyl 4-(4-ethoxyphenyl)-6-methyl-2-oxo-1,2,3,4-tetrahydropyrimidine-5-carboxylate	$C_{18}H_{25}N_3O_4$	347.40	31.25	++++	25.42	BN Acharya et al, Med Chem Res, 2015, 24, 1763-1775

**DCR73**	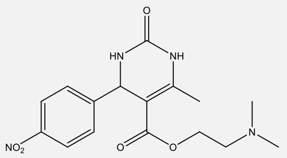	2-(dimethylamino)ethyl 6-methyl-4-(4-nitrophenyl)-2-oxo-1,2,3,4-tetrahydropyrimidine-5-carboxylate	$C_{16}H_{20}N_4O_5$	348.35	31.25	++++	24.54	BN Acharya et al, Med Chem Res, 2015, 24, 1763-1775
**DCR74**	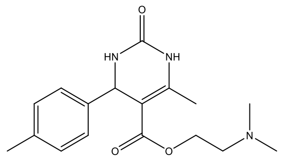	2-(dimethylamino)ethyl 6-methyl-4-(4-methylphenyl)-2-oxo-1,2,3,4-tetrahydropyrimidine-5-carboxylate	$C_{17}H_{23}N_3O_3$	317.38	31.25	++++	12.75	BN Acharya et al, Med Chem Res, 2015, 24, 1763-1775
**DCR75**	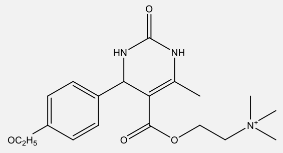	{2-[4-(4-ethoxyphenyl)-6-methyl-2-oxo-1,2,3,4-tetrahydropyrimidine-5-carbonyloxy]ethyl}trimethylazanium	$C_{19}H_{28}N_3O_4$	362.44	31.25	++++	10.21	BN Acharya et al, Med Chem Res, 2015, 24, 1763-1775
**DCR76**	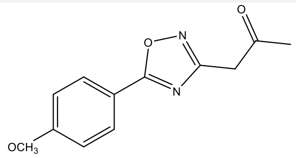	1-[5-(4-methoxyphenyl)-1,2,4-oxadiazol-3-yl]propan-2-one	$C_{12}H_{12}N_2O_3$	232.23	125	++++	19.42	
**DCR77**	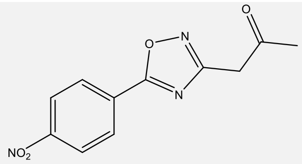	1-[5-(4-nitrophenyl)-1,2,4-oxadiazol-3-yl]propan-2-one	$C_{11}H_9N_3O_4$	247.20	125	++++	10.43	
**DCR78**	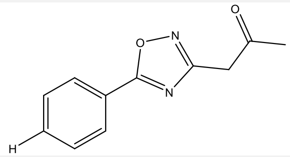	1-(5-phenyl-1,2,4-oxadiazol-3-yl)propan-2-one	$C_{11}H_{10}N_2O_2$	202.20	125	++++	17.32	
**DCR79**	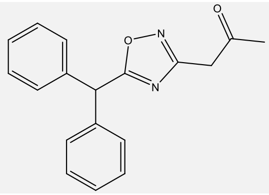	1-[5-(diphenylmethyl)-1,2,4-oxadiazol-3-yl]propan-2-one	$C_{18}H_{16}N_2O_2$	292.33	125	++++	16.42	
**DCR80**	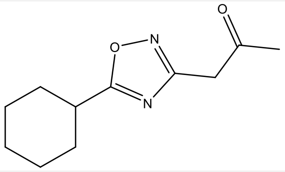	1-(5-cyclohexyl-1,2,4-oxadiazol-3-yl)propan-2-one	$C_{11}H_{16}N_2O_2$	208.25	125	++++	29.64	
**DCR81**	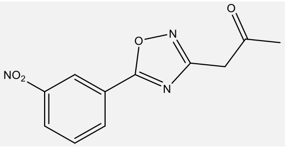	1-[5-(3-nitrophenyl)-1,2,4-oxadiazol-3-yl]propan-2-one	$C_{11}H_9N_3O_4$	247.20	125	++++	0	

**DCR82**	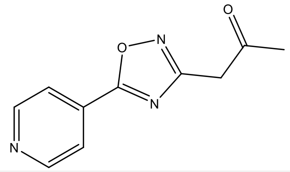	1-[5-(pyridin-4-yl)-1,2,4-oxadiazol-3-yl]propan-2-one	$C_{10}H_9N_3O_2$	203.19	125	++++	14.42	
**DCR83**	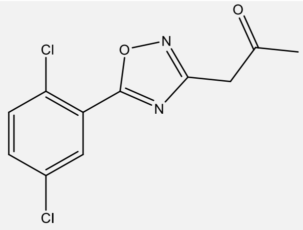	1-[5-(2,5-dichlorophenyl)-1,2,4-oxadiazol-3-yl]propan-2-one	$C_{11}H_8Cl_2N_2O_2$	271.09	230.55	++++	10.49	
**DCR84**	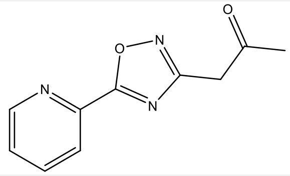	1-[5-(pyridin-2-yl)-1,2,4-oxadiazol-3-yl]propan-2-one	$C_{10}H_9N_3O_2$	203.19	125	++++	2.86	
**DCR85**	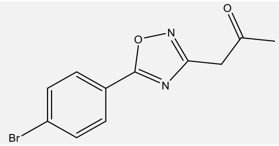	1-[5-(4-bromophenyl)-1,2,4-oxadiazol-3-yl]propan-2-one	$C_{11}H_9BrN_2O_2$	281.10	222.34	++++	10.38	
**DCR86**	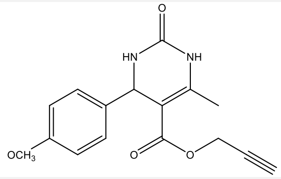	prop-2-yn-1-yl 4-(4-methoxyphenyl)-6-methyl-2-oxo-1,2,3,4-tetrahydropyrimidine-5-carboxylate	$C_{16}H_{16}N_2O_4$	300.30	208.12	++++	10.59	GBD Rao et al, RSC Adv, 2014, 4, 43321-43325
**DCR87**	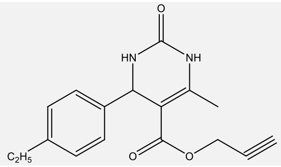	prop-2-yn-1-yl 4-(4-ethylphenyl)-6-methyl-2-oxo-1,2,3,4-tetrahydropyrimidine-5-carboxylate	$C_{17}H_{18}N_2O_3$	298.33	104.58	++++	11.61	GBD Rao et al, RSC Adv, 2014, 4, 43321-43325
**DCR88**	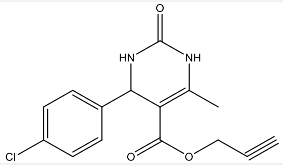	prop-2-yn-1-yl 4-(4-chlorophenyl)-6-methyl-2-oxo-1,2,3,4-tetrahydropyrimidine-5-carboxylate	$C_{15}H_{13}ClN_2O_3$	304.72	205.1	++++	11.05	GBD Rao et al, RSC Adv, 2014, 4, 43321-43325
**DCR89**	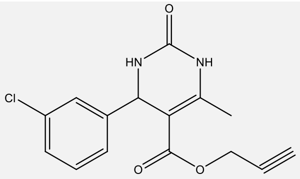	prop-2-yn-1-yl 4-(3-chlorophenyl)-6-methyl-2-oxo-1,2,3,4-tetrahydropyrimidine-5-carboxylate	$C_{15}H_{13}ClN_2O_3$	304.72	205.1	++++	10.94	GBD Rao et al, RSC Adv, 2014, 4, 43321-43325
**DCR90**	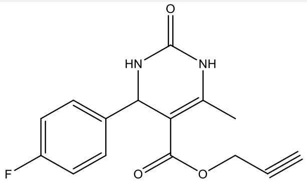	prop-2-yn-1-yl 4-(4-fluorophenyl)-6-methyl-2-oxo-1,2,3,4-tetrahydropyrimidine-5-carboxylate	$C_{15}H_{13}FN_2O_3$	288.27	433.62	++++	12.59	GBD Rao et al, RSC Adv, 2014, 4, 43321-43325

**DCR91**	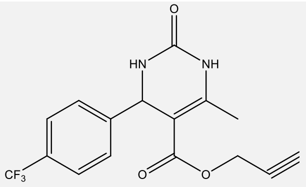	prop-2-yn-1-yl 6-methyl-2-oxo-4-[4-(trifluoromethyl)phenyl]-1,2,3,4-tetrahydropyrimidine-5-carboxylate	$C_{16}H_{13}F_3N_2O_3$	338.28	369.52	++++	13.12	GBD Rao et al, RSC Adv, 2014, 4, 43321-43325
**DCR92**	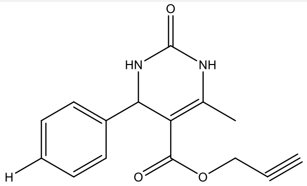	prop-2-yn-1-yl 6-methyl-2-oxo-4-phenyl-1,2,3,4-tetrahydropyrimidine-5-carboxylate	$C_{15}H_{14}N_2O_3$	270.28	462.48	++++	12.8	GBD Rao et al, RSC Adv, 2014, 4, 43321-43325
**DCR93**	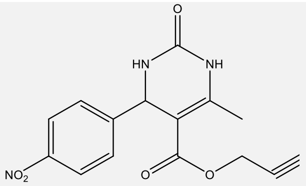	prop-2-yn-1-yl 6-methyl-4-(4-nitrophenyl)-2-oxo-1,2,3,4-tetrahydropyrimidine-5-carboxylate	$C_{15}H_{13}N_3O_5$	315.28	315.28	++++	12.17	GBD Rao et al, RSC Adv, 2014, 4, 43321-43325
**DCR94**	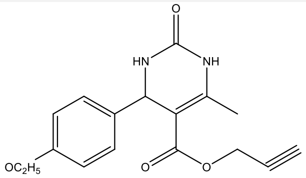	prop-2-yn-1-yl 4-(4-ethoxyphenyl)-6-methyl-2-oxo-1,2,3,4-tetrahydropyrimidine-5-carboxylate	$C_{17}H_{18}N_2O_4$	314.33	198.83	++++	19.5	GBD Rao et al, RSC Adv, 2014, 4, 43321-43325
**DCR95**	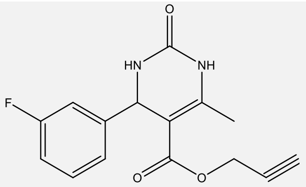	prop-2-yn-1-yl 4-(3-fluorophenyl)-6-methyl-2-oxo-1,2,3,4-tetrahydropyrimidine-5-carboxylate	$C_{15}H_{13}FN_2O_3$	288.27	433.62	++++	11.54	GBD Rao et al, RSC Adv, 2014, 4, 43321-43325
**DCR96**	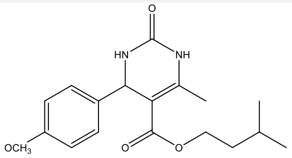	3-methylbutyl 4-(4-methoxyphenyl)-6-methyl-2-oxo-1,2,3,4-tetrahydropyrimidine-5-carboxylate	$C_{18}H_{24}N_2O_4$	332.39	376.06	++++	11.71	GBD Rao et al, RSC Adv, 2014, 4, 43321-43325
**DCR97**	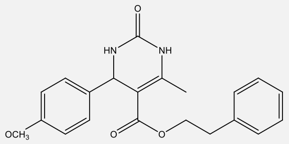	2-phenylethyl 4-(4-methoxyphenyl)-6-methyl-2-oxo-1,2,3,4-tetrahydropyrimidine-5-carboxylate	$C_{21}H_{22}N_2O_4$	366.41	170.57	++++	11.19	GBD Rao et al, RSC Adv, 2014, 4, 43321-43325
**DCR98**	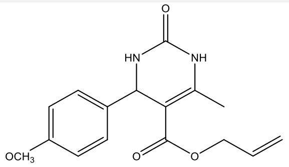	prop-2-en-1-yl 4-(4-methoxyphenyl)-6-methyl-2-oxo-1,2,3,4-tetrahydropyrimidine-5-carboxylate	$C_{16}H_{18}N_2O_4$	302.32	413.46	++++	12.66	GBD Rao et al, RSC Adv, 2014, 4, 43321-43325

**DCR99**	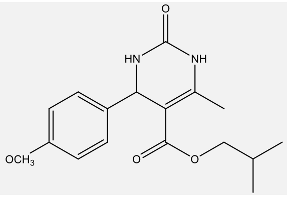	2-methylpropyl 4-(4-methoxyphenyl)-6-methyl-2-oxo-1,2,3,4-tetrahydropyrimidine-5-carboxylate	$C_{17}H_{22}N_2O_4$	318.36	392.64	++++	11.29	GBD Rao et al, RSC Adv, 2014, 4, 43321-43325
**DCR100**	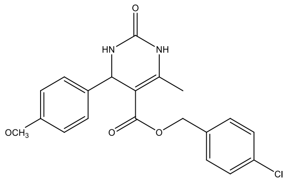	(4-chlorophenyl)methyl 4-(4-methoxyphenyl)-6-methyl-2-oxo-1,2,3,4-tetrahydropyrimidine-5-carboxylate	$C_{20}H_{19}ClN_2O_4$	386.82	323.15	++++	12.84	GBD Rao et al, RSC Adv, 2014, 4, 43321-43325
**DCR101**	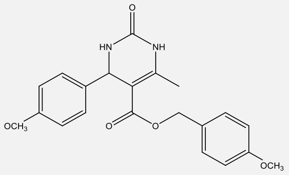	4-methoxybenzyl 4-(4-methoxyphenyl)-6-methyl-2-oxo-1,2,3,4-tetrahydropyrimidine-5-carboxylate	$C_{21}H_{22}N_2O_5$	382.40	250	++++	7.02	GBD Rao et al, RSC Adv, 2014, 4, 43321-43325
**DCR102**	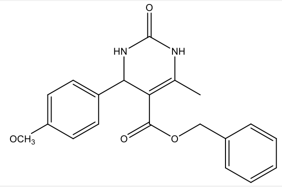	benzyl 4-(4-methoxyphenyl)-6-methyl-2-oxo-1,2,3,4-tetrahydropyrimidine-5-carboxylate	$C_{20}H_{20}N_2O_4$	352.38	250	++++	8.11	GBD Rao et al, RSC Adv, 2014, 4, 43321-43325
**DCR103**	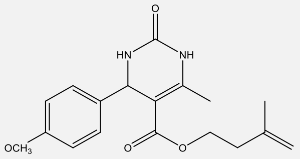	3-methylbut-3-en-1-yl 4-(4-methoxyphenyl)-6-methyl-2-oxo-1,2,3,4-tetrahydropyrimidine-5-carboxylate	$C_{18}H_{22}N_2O_4$	330.37	250	++++	21.11	GBD Rao et al, RSC Adv, 2014, 4, 43321-43325
**DCR104**	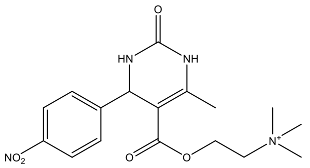	*N*,*N*,*N*-trimethyl-2-({[6-methyl-4-(4-nitrophenyl)-2-oxo-1,2,3,4-tetrahydropyrimidin-5-yl]carbonyl}oxy)ethanaminium	$C_{17}H_{23}N_4O_5$	363.38	125	++++	9.09	BN Acharya et al, Med Chem Res, 2015, 24, 1763-1775
**DCR105**	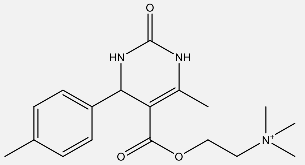	*N*,*N*,*N*-trimethyl-2-({[6-methyl-4-(4-methylphenyl)-2-oxo-1,2,3,4-tetrahydropyrimidin-5-yl]carbonyl}oxy)ethanaminium	$C_{18}H_{26}N_3O_3$	332.41	125	++++	2.45	BN Acharya et al, Med Chem Res, 2015, 24, 1763-1775
**DCR106**	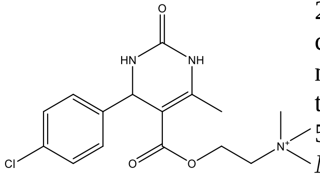	2-({[4-(4-chlorophenyl)-6-methyl-2-oxo-1,2,3,4-tetrahydropyrimidin-5-yl]carbonyl}oxy)-*N*,*N*,*N*-	$C_{17}H_{23}ClN_3O_3$	352.83	62.5	++++	3.33	BN Acharya et al, Med Chem Res, 2015, 24, 1763-1775

		trimethylethanaminium						
**DCR107**	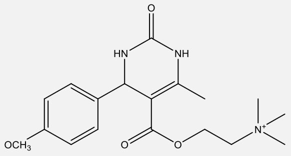	{2-[4-(4-methoxyphenyl)-6-methyl-2-oxo-1,2,3,4-tetrahydropyrimidine-5-carbonyloxy]ethyl}trimethylazanium	$C_{18}H_{26}N_3O_4$	348.41	62.5	++++	13.22	BN Acharya et al, Med Chem Res, 2015, 24, 1763-1775
**DCR108**	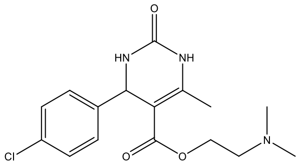	2-(dimethylamino)ethyl 4-(4-chlorophenyl)-6-methyl-2-oxo-1,2,3,4-tetrahydropyrimidine-5-carboxylate	$C_{16}H_{20}ClN_3O_3$	337.80	62.5	++++	13.99	BN Acharya et al, Med Chem Res, 2015, 24, 1763-1775
**DCR109**	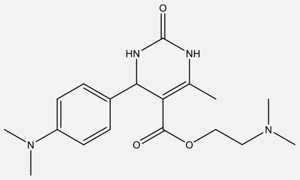	2-(dimethylamino)ethyl 4-[4-(dimethylamino)phenyl]-6-methyl-2-oxo-1,2,3,4-tetrahydropyrimidine-5-carboxylate	$C_{18}H_{26}N_4O_3$	346.42	62.5	++++	15.11	BN Acharya et al, Med Chem Res, 2015, 24, 1763-1775
**DCR110**	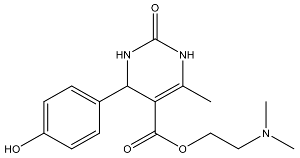	2-(dimethylamino)ethyl 4-(4-hydroxyphenyl)-6-methyl-2-oxo-1,2,3,4-tetrahydropyrimidine-5-carboxylate	$C_{16}H_{21}N_3O_4$	319.35	62.5	++++	12.01	BN Acharya et al, Med Chem Res, 2015, 24, 1763-1775
**DCR111**	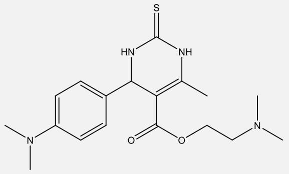	2-(dimethylamino)ethyl 4-[4-(dimethylamino)phenyl]-6-methyl-2-sulfanylidene-1,2,3,4-tetrahydropyrimidine-5-carboxylate	$C_{18}H_{26}N_4O_2S$	362.48	62.5	++++	10.08	BN Acharya et al, Med Chem Res, 2015, 24, 1763-1775
**DCR112**	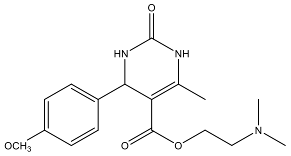	2-(dimethylamino)ethyl 4-(4-methoxyphenyl)-6-methyl-2-oxo-1,2,3,4-tetrahydropyrimidine-5-carboxylate	$C_{17}H_{23}N_3O_4$	333.38	62.5	++++	2.09	BN Acharya et al, Med Chem Res, 2015, 24, 1763-1775

**DCR113**	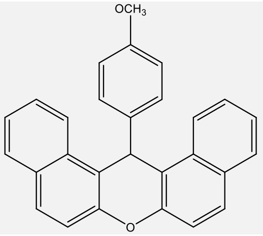	2-(4-methoxyphenyl)-13-oxapentacyclo[12.8.0.$0^{3,12}.0^{4,9}.0^{17,22}$]docosa-1(14),3,5,7,9,11,15,17(22),18,20-decaene	$C_{28}H_{20}O_2$	388.45	31.25	++++	5.04	GBD Rao et al, Tetrahedron Lett. 2012, 53, 2741-2744
**DCR114**	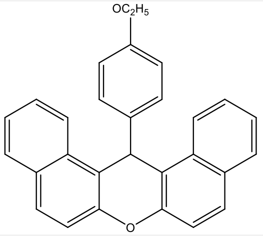	2-(4-ethoxyphenyl)-13-oxapentacyclo[12.8.0.$0^{3,12}.0^{4,9}.0^{17,22}$]docosa-1(14),3,5,7,9,11,15,17(22),18,20-decaene	$C_{29}H_{22}O_2$	402.48	31.25	++++	4.05	GBD Rao et al, Tetrahedron Lett. 2012, 53, 2741-2744
**DCR115**	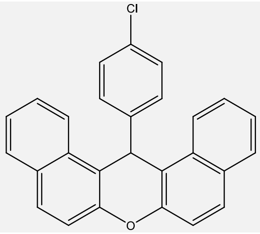	2-(4-chlorophenyl)-13-oxapentacyclo[12.8.0.$0^{3,12}.0^{4,9}.0^{17,22}$]docosa-1(14),3,5,7,9,11,15,17(22),18,20-decaene	$C_{27}H_{17}ClO$	392.87	125	++++	17.1	GBD Rao et al, Tetrahedron Lett. 2012, 53, 2741-2744
**DCR116**	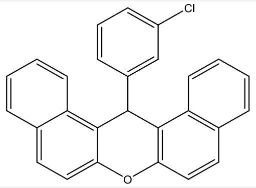	2-(3-chlorophenyl)-13-oxapentacyclo[12.8.0.$0^{3,12}.0^{4,9}.0^{17,22}$]docosa-1(14),3,5,7,9,11,15,17(22),18,20-decaene	$C_{27}H_{17}ClO$	392.87	31.25	++++	10.84	GBD Rao et al, Tetrahedron Lett. 2012, 53, 2741-2744
**DCR117**	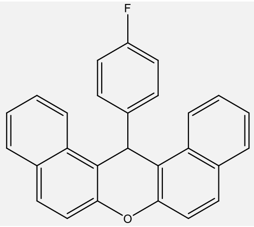	2-(4-fluorophenyl)-13-oxapentacyclo[12.8.0.$0^{3,12}.0^{4,9}.0^{17,22}$]docosa-1(14),3,5,7,9,11,15,17(22),18,20-decaene	$C_{27}H_{17}FO$	376.42	125	++++	14.01	GBD Rao et al, Tetrahedron Lett. 2012, 53, 2741-2744
**DCR118**	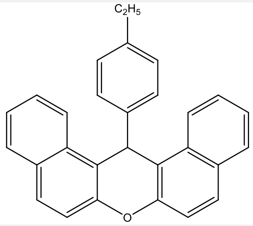	2-(4-ethylphenyl)-13-oxapentacyclo[12.8.0.$0^{3,12}.0^{4,9}.0^{17,22}$]docosa-1(14),3,5,7,9,11,15,17(22),18,20-decaene	$C_{29}H_{22}O$	386.48	31.25	++++	14.23	GBD Rao et al, Tetrahedron Lett. 2012, 53, 2741-2744
**DCR119**	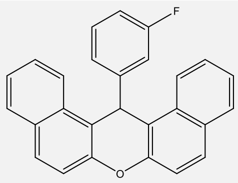	2-(3-fluorophenyl)-13-oxapentacyclo[12.8.0.$0^{3,12}.0^{4,9}.0^{17,22}$]docosa-1(14),3,5,7,9,11,15,17(22),18,20-decaene	$C_{27}H_{17}FO$	376.42	31.25	++++	18.74	GBD Rao et al, Tetrahedron Lett. 2012, 53, 2741-2744
**DCR120**	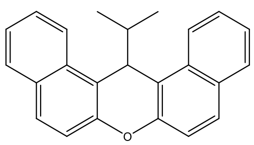	2-(propan-2-yl)-13-oxapentacyclo[12.8.0.$0^{3,12}.0^{4,9}.0^{17,22}$]docosa-1(14),3,5,7,9,11,15,17(22),18,20-decaene	$C_{24}H_{20}O$	324.41	31.25	++++	19.24	GBD Rao et al, Tetrahedron Lett. 2012, 53, 2741-2744

**DCR121**	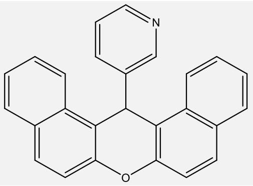	3-{13-oxapentacyclo[12.8.0.0^{3,12}.0^{4,9}.0^{17,22}]docosa-1(14),3,5,7,9,11,15,17(22),18,20-decaen-2-yl}pyridine	$C_{26}H_{17}NO$	359.41	31.25	++++	8.03	GBD Rao et al, Tetrahedron Lett. 2012, 53, 2741-2744
**DCR122**	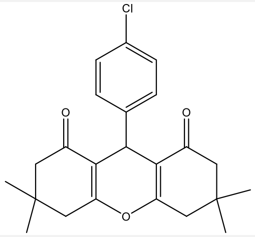	9-(4-chlorophenyl)-3,3,6,6-tetramethyl-2,3,4,5,6,7,8,9-octahydro-1H-xanthene-1,8-dione	$C_{23}H_{25}ClO_3$	384.89	31.25	++++	11.09	GBD Rao et al, Heterocyclic Lett., 2012, 2, 411-418.
**DCR123**	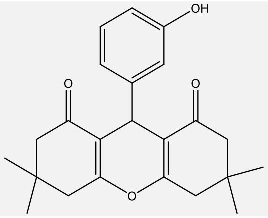	9-(3-hydroxyphenyl)-3,3,6,6-tetramethyl-2,3,4,5,6,7,8,9-octahydro-1H-xanthene-1,8-dione	$C_{23}H_{26}O_4$	366.45	125	++++	12.01	GBD Rao et al, Heterocyclic Lett., 2012, 2, 411-418.
**DCR124**	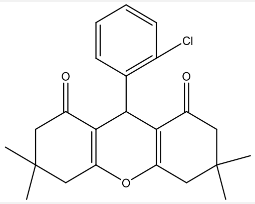	9-(2-chlorophenyl)-3,3,6,6-tetramethyl-2,3,4,5,6,7,8,9-octahydro-1H-xanthene-1,8-dione	$C_{23}H_{25}ClO_3$	384.89	125	++++	11.05	GBD Rao et al, Heterocyclic Lett., 2012, 2, 411-418.
**DCR125**	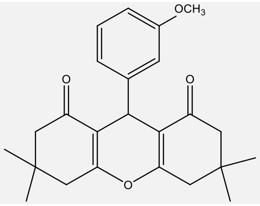	9-(3-methoxyphenyl)-3,3,6,6-tetramethyl-2,3,4,5,6,7,8,9-octahydro-1H-xanthene-1,8-dione	$C_{24}H_{28}O_4$	380.47	125	++++	11.19	GBD Rao et al, Heterocyclic Lett., 2012, 2, 411-418.
**DCR126**	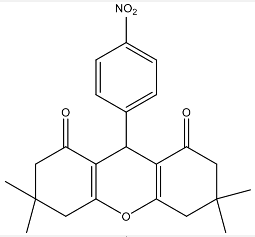	3,3,6,6-tetramethyl-9-(4-nitrophenyl)-2,3,4,5,6,7,8,9-octahydro-1H-xanthene-1,8-dione	$C_{23}H_{25}NO_5$	395.44	15.6	++++	17.99	GBD Rao et al, Heterocyclic Lett., 2012, 2, 411-418.
**DCR127**	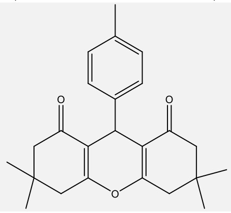	3,3,6,6-tetramethyl-9-(4-methylphenyl)-2,3,4,5,6,7,8,9-octahydro-1H-xanthene-1,8-dione	$C_{24}H_{28}O_3$	364.47	125	++++	10.21	GBD Rao et al, Heterocyclic Lett., 2012, 2, 411-418.
**DCR128**	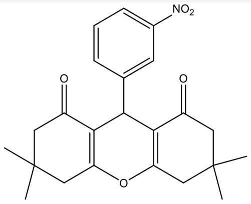	9-(4-methoxyphenyl)-3,3,6,6-tetramethyl-2,3,4,5,6,7,8,9-octahydro-1H-xanthene-1,8-dione	$C_{24}H_{28}O_4$	380.47	125	++++	11.01	GBD Rao et al, Heterocyclic Lett., 2012, 2, 411-418.

**DCR129**	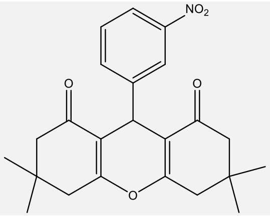	3,3,6,6-tetramethyl-9-(3-nitrophenyl)-2,3,4,5,6,7,8,9-octahydro-1H-xanthene-1,8-dione	$C_{23}H_{25}NO_5$	395.44	31.25	++++	16.85	GBD Rao et al, Heterocyclic Lett., 2012, 2, 411-418.
**DCR130**	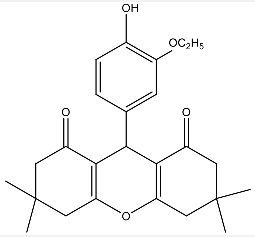	9-(3-ethoxy-4-hydroxyphenyl)-3,3,6,6-tetramethyl-2,3,4,5,6,7,8,9-octahydro-1H-xanthene-1,8-dione	$C_{25}H_{30}O_5$	410.50	15.6	++++	17.21	GBD Rao et al, Heterocyclic Lett., 2012, 2, 411-418.
**DCR131**	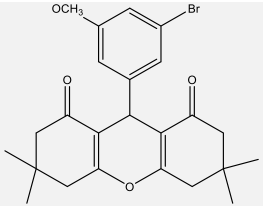	9-(3-bromo-5-methoxyphenyl)-3,3,6,6-tetramethyl-2,3,4,5,6,7,8,9-octahydro-1H-xanthene-1,8-dione	$C_{24}H_{27}BrO_4$	459.37	125	++++	8.01	GBD Rao et al, Heterocyclic Lett., 2012, 2, 411-418.
**DCR132**	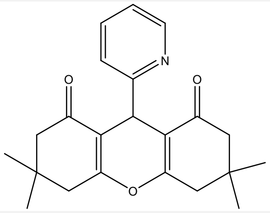	3,3,6,6-tetramethyl-9-(pyridin-2-yl)-2,3,4,5,6,7,8,9-octahydro-1H-xanthene-1,8-dione	$C_{22}H_{25}NO_3$	351.43	125	++++	9.06	GBD Rao et al, Heterocyclic Lett., 2012, 2, 411-418.
**DCR133**	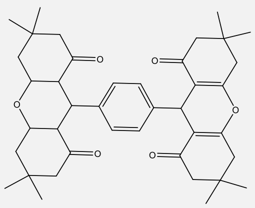	3,3,6,6-tetramethyl-9-[4-(3,3,6,6-tetramethyl-1,8-dioxo-dodecahydro-1H-xanthen-9-yl)phenyl]-2,3,4,5,6,7,8,9-octahydro-1H-xanthene-1,8-dione	$C_{40}H_{50}O_6$	626.82	125	++++	11.14	GBD Rao et al, Heterocyclic Lett., 2012, 2, 411-418.
**DCR134**	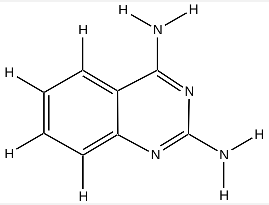	Quinazoline-2,4-diamine	C8H8N4	160.18	150	+	75.9	N Shelke et al, RSC Adv. 2015, 5, 31226-31230
**DCR135**	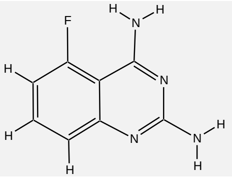	5-Fluoro-quinazoline-2,4-diamine	C8H7FN4	178.17	250	++	61	N Shelke et al, RSC Adv. 2015, 5, 31226-31230
**DCR136**	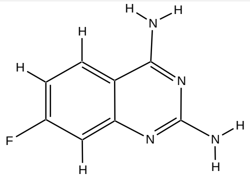	7-Fluoro-quinazoline-2,4-diamine	C8H7FN4	178.17	250	++	54	N Shelke et al, RSC Adv. 2015, 5, 31226-31230

**DCR137**	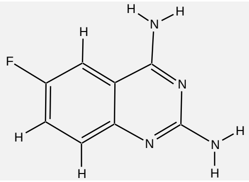	6-Fluoro-quinazoline-2,4-diamine	C8H7FN4	178.17	300	+	90.3	N Shelke et al, RSC Adv. 2015, 5, 31226-31230
**DCR138**	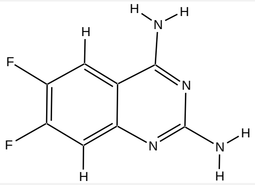	6,7-Difluoro-quinazoline-2,4-diamine	C8H6F2N4	196.16	250	++	51	N Shelke et al, RSC Adv. 2015, 5, 31226-31230
**DCR139**	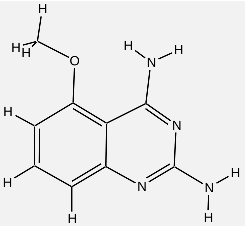	5-Methoxy-quinazoline-2,4-diamine	C9H10N4O	190.20	250	++	57	N Shelke et al, RSC Adv. 2015, 5, 31226-31230
**DCR140**	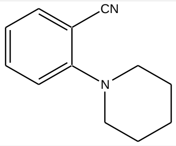	2-Piperidin-1-yl-benzonitrile	C12H14N2	186.25	250	++++	14.97	N Shelke et al, RSC Adv. 2015, 5, 31226-31230
**DCR141**	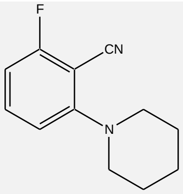	2-Fluoro-6-piperidin-1-yl-benzonitrile	C12H13FN2	204.24	125	++++	19.24	N Shelke et al, RSC Adv. 2015, 5, 31226-31230
**DCR142**	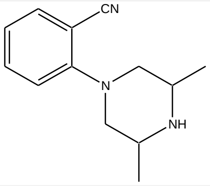	2-(3,5-Dimethyl-piperazin-1-yl)-benzonitrile	C13H17N3	215.29	125	++++	13.08	N Shelke et al, RSC Adv. 2015, 5, 31226-31230
**DCR143**	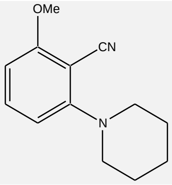	2-Methoxy-6-piperidin-1-yl-benzonitrile	C13H16N2O	216.28	125	++++	17.42	N Shelke et al, RSC Adv. 2015, 5, 31226-31230
**DCR144**	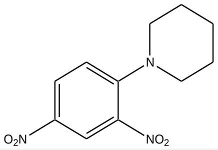	1-(2,4-Dinitro-phenyl)-piperidine	C11H13N3O4	251.24	125	++++	25.21	N Shelke et al, RSC Adv. 2015, 5, 31226-31230
**DCR145**	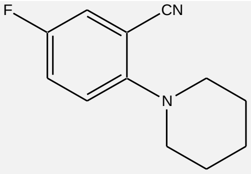	5-Fluoro-2-piperidin-1-yl-benzonitrile	C12H13FN2	204.24	125	++++	0	N Shelke et al, RSC Adv. 2015, 5, 31226-31230

**DCR146**	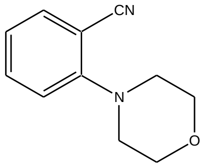	2-Morpholin-4-yl-benzonitrile	C11H12N2O	188.23	250	++++	-3.98	N Shelke et al, RSC Adv. 2015, 5, 31226-31230
**DCR147**	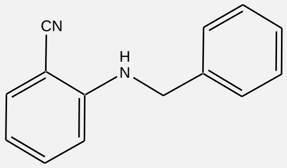	2-Benzylamino-benzonitrile	C14H12N2	208.26	125	++++	17.97	N Shelke et al, RSC Adv. 2015, 5, 31226-31230
**DCR148**	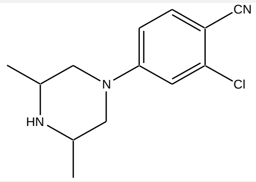	2-Chloro-4-(3,5-dimethyl-piperazin-1-yl)-benzonitrile	C13H16ClN3	249.74	125	++++	14.42	
**DCR149**	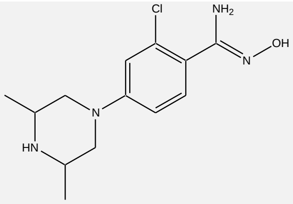	2-Chloro-4-(3,5-dimethyl-piperazin-1-yl)-N-hydroxy-benzamidine	C13H19ClN4O	282.77	125	++++	-21.8	
**DCR150**	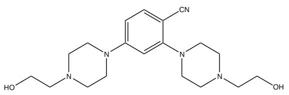	2,4-Bis-[4-(2-hydroxy-ethyl)-piperazin-1-yl]-benzonitrile	C19H29N5O2	359.47	62.5	++++	18.09	

*Maximum nontoxic dose (MNTD) of all the compounds were calculated on Vero cells.

**Arbitrary score given on the basis of visualization of the cytopathic effect (CPE) at >48 hpi: >60% (++++), 60-50% (+++), 50-30% (++), and <30% (+).

***Cell viability at 72 hpi as assessed by MTT assay.
